# Health information management and perceptions of the quality of care for children with tracheotomy: A qualitative study

**DOI:** 10.1186/1472-6963-11-117

**Published:** 2011-05-23

**Authors:** Jay G Berry, Donald A Goldmann, Kenneth D Mandl, Heather Putney, David Helm, Jane O'Brien, Richard Antonelli, Robin M Weinick

**Affiliations:** 1 Division of General Pediatrics, Children's Hospital, Boston, MA, USA; 2 Institute for Healthcare Improvement, Cambridge, MA, USA; 3 Children's Hospital Informatics Program, Children's Hospital, Boston, MA, USA; 4 Developmental Medicine Center, Children's Hospital, Boston, MA, USA; 5 Franciscan Hospital for Children, Brighton, MA, USA; 6 Children's Hospital Integrated Care Organization, Children's Hospital, Boston, MA, USA; 7 The RAND Corporation, Arlington, VA, USA

## Abstract

**Background:**

Children with tracheotomy receive health care from an array of providers within various hospital and community health system sectors. Previous studies have highlighted substandard health information exchange between families and these sectors. The aim of this study was to investigate the perceptions and experiences of parents and providers with regard to health information management, care plan development and coordination for children with tracheotomy, and strategies to improve health information management for these children.

**Methods:**

Individual and group interviews were performed with eight parents and fifteen healthcare (primary and specialty care, nursing, therapist, equipment) providers of children with tracheotomy. The primary tracheotomy-associated diagnoses for the children were neuromuscular impairment (n = 3), airway anomaly (n = 2) and chronic lung disease (n = 3). Two independent reviewers conducted deep reading and line-by-line coding of all transcribed interviews to discover themes associated with the objectives.

**Results:**

Children with tracheotomy in this study had healthcare providers with poorly defined roles and responsibilities who did not actively communicate with one another. Providers were often unsure where to find documentation relating to a child's tracheotomy equipment settings and home nursing orders, and perceived that these situations contributed to medical errors and delayed equipment needs. Parents created a home record that was shared with multiple providers to track the care that their children received but many considered this a burden better suited to providers. Providers benefited from the parent records, but questioned their accuracy regarding critical tracheotomy care plan information such as ventilator settings. Parents and providers endorsed potential improvement in this environment such as a comprehensive internet-based health record that could be shared among parents and providers, and between various clinical sites.

**Conclusions:**

Participants described disorganized tracheotomy care and health information mismanagement that could help guide future investigations into the impact of improved health information systems for children with tracheotomy. Strategies with the potential to improve tracheotomy care delivery could include defined roles and responsibilities for tracheotomy providers, and improved organization and parent support for maintenance of home-based tracheotomy records with web-based software applications, personal health record platforms and health record data authentication techniques.

## Background

Tracheotomy is being increasingly performed on children with complex chronic medical conditions including neurological impairment and chronic lung disease [1]. The children's medical complexity derives from the use of multiple medications, dependence on respiratory equipment requiring frequent adjustment, and heavy reliance on home care-givers with substantial clinical expertise to maintain health [2]. Children with tracheotomy receive health care from an array of providers including otolaryngologists, pulmonologists, primary care, rehabilitation care, home and school nursing, therapists and equipment specialists [3,4]. However, services delivered by these providers are often spread among multiple locations.

Previous studies have highlighted the complex interface between families and multiple health system sectors for children with special healthcare needs (CSHCN), including children with tracheotomy. Challenges that providers, patients and families experience include poor availability, accessibility and exchange of medical information and the absence of coordinated care [5-9]. This may impede a provider's ability to recognize and meet a child's healthcare needs, particularly during an acute illness, and could place children at risk of receiving sub-optimal quality of care, resulting in poor health outcomes.

One strategy to overcome these challenges is a medical home concept offering a central, accessible medical record containing all pertinent medical information relating to hospitalization and specialty care [10]. Some components of the medical home concept have been achieved for several CSHCN but the full model that includes a central, accessible record is not readily available for the majority of patients and their families [11]. The multidisciplinary medical needs of children with tracheotomy may preclude a single pediatric practice sustaining a centralized health record, and it is unlikely that such patients will have access to a central health record in other health care settings [12].

There are emerging strategies to facilitate health information management and exchange across different healthcare sectors, including healthcare reform initiatives promoting meaningful use of electronic health records (EHR). For example, patients, families and outpatient providers may view selected parts of a patient's hospital-based EHR using an internet-based portal [13]. However, few systems have been customized for pediatric patients, and little information is available for informatic architects and software programmers concerning how children with complex medical needs such as tracheotomy could use and benefit from these strategies [14].

In response to these concerns, this study addresses the following questions: (1) How does health information management and sharing contribute to the perceptions of quality of care received by children with tracheotomy? (2) How could the exchange of health information among various providers be improved? To address these questions, the perceptions and experiences of parents and providers with regard to health information management, care coordination and care plan development for children with tracheotomy were investigated. Furthermore, strategies to improve health information management for these children were explored.

## Methods

### Study Design

Semi-structured individual and group interviews of parents and healthcare providers of children with tracheotomy were conducted. This qualitative method allows more time to be spent with participants, and therefore detailed experiences and perceptions can be acquired that could be missed using quantitative surveys [15,16]. The Children's Hospital, Boston (CHB) and Franciscan Hospital for Children (FHC) Institutional Review Boards (# X09-06-0320) approved this study in compliance with the Helsinki Declaration.

Individual interviews were conducted for participant convenience (e.g., if a participant could not attend a scheduled group interview) and preference (e.g., if a participant wished to be interviewed privately to ensure anonymity and confidentiality). Group interviews were conducted to elicit important information from conversation among participants that would not occur with individual interviews. Open-ended questions based on constructs from the Chronic Care and medical home models were asked [10,17]. These constructs include centralized access to and sharing of the child's health records, patient and family-centered care, care coordination and care plan development.

### Study Participants

Eligible children were aged 2-18 years, had an existing tracheotomy for a minimum of one year, and had at least one medical encounter with a healthcare provider at the CHB or FHC with a tracheotomy billing code (31.1 or 31.2; V44.0, V55.0, or 519.00-519.09) [18,19]. Children aged ≥2 years were included as the families had gained experience in terms of navigating their child's health system. FHC is a pediatric rehabilitation hospital for children with special healthcare needs in Boston, MA. CHB is a freestanding acute-care children's hospital that offers primary, specialty and hospital care. We performed purposive sampling by reviewing the list of eligible participants and recruiting those likely to have information-rich cases [20].

Parents of subjects were identified through chart review and recruited by phone or email. Written informed consent was obtained for all enrolled participants. Parents (n = 8) accepted enrollment and gave consent to approach their child's healthcare providers for participation in the study. Each parent came from a different family and all parents interviewed were mothers. Enrolled parents had children with tracheotomy aged between 6 and 18 years. The primary tracheotomy-associated diagnoses for these children were neuromuscular impairment (n = 3), airway anomaly (n = 2) and chronic lung disease (n = 3), and 50% of these children required daily mechanical ventilation; the remaining children utilized oxygen or humidified air. All children lived at home and had visited a primary care physician at least once within the previous year.

Tracheotomy providers identified by parents were eligible for study participation if they had delivered clinical care to one of the children within the past year, and their names were verified within the CHB or FHC medical record. Tracheotomy providers of all types from all institutions and clinics in the greater-Boston area were approached for enrollment in the study. Fifteen providers agreed to participate in the study and one provider declined. Eight providers were physicians: five were primary care pediatricians and three were pediatric specialists (otolaryngology, pulmonology and intensive care). The remaining providers (n = 7) were nurses (primary care, home, school and hospital), respiratory therapists and equipment vendors. All patients had at least one relationship with an enrolled provider. One patient had multiple relationships (i.e. one child was cared for by a home nurse and a respiratory therapist).

### Data Collection Procedures

Study team members, JGB and HP, conducted separate individual and group interviews with parents and providers. The authors had no clinical relationship with any child with a tracheotomy whose parents and providers were enrolled in the study. Individual parent interviews averaged 60 minutes (n = 3), and the group interview with parents (n = 5 participants) lasted 150 minutes. Two parent interviews were conducted in an out-patient clinic office setting and the remaining individual parent interviews and group interviews were conducted in family homes. Individual provider interviews (n = 8) averaged 30 minutes, and the group provider interview (n = 7 participants) lasted 60 minutes; these were conducted in the outpatient clinic office setting. Non-directive interviewing techniques were utilized to minimize interviewer-related errors and the potential to bias answers given by participants. Additional file 1, table S1 presents the core questions used to guide the discussions [15,16]. Individual and group interviews produced 11 cumulative hours of audio-taped data with 80,840 transcribed words.

### Qualitative Analysis

Thematic qualitative analysis was performed to investigate and describe parent and provider experiences and perceptions. Two independent reviewers (JGB and HP) coded the parent and provider transcripts independently to identify key points discussed by the interviewees [21,22]. A third author (RMW) triangulated the coding strategy and arbitrated discrepancies between the two coders. Line-by-line coding was performed at the beginning of the study for rapid generation of concepts without inducing coder bias [22]. Grounded theory [23] was used to group related concepts and NVIVO 8 [24] was utilized to form themes from these related concepts. The themes served as a basis for providing a greater understanding of health information management for children with tracheotomy. Illustrative quotations are presented that reflect supportive and deviant cases, as well as alternative explanations of the theory.

Several data verification procedures including concurrent data collecting and analyzing, idea reconfirmation (during the process) and member checks (post-hoc), were carried out to confirm the reliability of the data generated [25]. With member checks, parent experiences were corroborated by tracheotomy clinicians who verified the experiences. Furthermore, the results were verified with four parents in a two hour didactic group meeting. Participant enrollment was discontinued once theme saturation was observed. Theme saturation was determined by completing data analysis on six parent and twelve provider interviews. Data were then analyzed from two additional parent and three additional provider interviews, and no new themes were discovered.

## Results

### 1. Parents and providers felt that no child with tracheotomy had a medical record containing comprehensive health information from all of the child's providers in one central location

Children's health information was described as being scattered across multiple providers in various settings and institutions without a clear "home" for their aggregated health information. Parents and providers noted the absence of a care plan summary within the child's existing health records presenting the child's active medications, tracheotomy equipment settings, pertinent past medical history and acute-illness instructions in one readily-accessible document. Rather, "bits and pieces" of this health information were fragmented within multiple medical records residing in multiple hospital and community locations.

#### Pediatric Intensivist

"Tracheotomy patients may come to the intensive care unit with a chief complaint and a brief synopsis, but not necessarily a comprehensive care regimen. They often come with a med list, but not necessarily anything about medical devices, whether it be trach or otherwise. So you're sort of left in a bind."

#### Parent

"This summer we went to a sleep study and had to be at [Hospital 2] instead of [our primary hospital]. The flow of information between hospitals is really difficult. So having to get people up to speed on who [my child] is and all his background information can be really difficult. So that's what's really tough...going from hospital to hospital and having that kind of flow of information... because that really bogs people down with sharing information and hospitals not wanting to do things unless they have [my child's] clear [health] information. It just gets really cumbersome and then you feel like you have to give...all of [my child's] information at once: the whole history. [Providers] get really scared of working with [my child]."

### 2. Parents and providers felt no designated provider was "in charge" of the child's health information with a key responsibility for coordination

Primary care providers were nervous about maintaining and taking responsibility for a comprehensive record because the child's health information, particularly regarding equipment settings and diet plans, was frequently updated by other providers. Parents and providers felt that the lack of a comprehensive health record was associated with their perceptions of sub-standard quality of care that the children received. Without a comprehensive record, parents and providers described experiences of delayed care, fragmented treatment planning and inaccessible or out-dated equipment and medical orders. These experiences were salient during care transitions such as when a child was leaving hospital to go home, or when a child needed help during an acute illness.

#### Otolaryngologist

"It's hard to be responsible for something when you're not aware of it and the information is not readily forthcoming... I think the [tracheotomy health] information isn't always readily available and there is no good home for it."

#### Pediatric Intensivist

"[Children with tracheostomy] often have so many providers involved that it's often difficult to get one person to assume primary responsibility [for their health information]. And as a result, the parents don't necessarily know who to go to [for information], or they may be getting inconsistent messages. As the provider, if there's not a point person, it can be very difficult simply because you don't know who to contact and what information is the most up-to-date if you see something that comes up. They're seeing providers at different institutions that aren't sharing information. And [information] systems may not be compatible. And so that makes it all that much more challenging."

#### School Nurse

"I quite honestly don't know who's holding the [health] information...The community [nurses] are having a hard time getting the [tracheotomy] orders from the primary care folks or the providers at the hospital... [I have one child that] didn't have [home] orders for eighteen months and they didn't have orders at school [for five months]; I think that if the information isn't transferred, you can have errors around kids on vents and then settings can be wrong. If updates aren't given, and the families aren't telling nurses that things are changing, then people are operating off of old orders. I think there can be a lot of errors that can occur."

#### Equipment Vendor

"[One child] is complex with a ventriculostomy and a trach and [ventilation needs]. She has multiple specialists. Who is our 'go to person' when it's time one year from now to get renewed authorizations for everything? We find ourselves scrambling sometimes...because we'll send [the prescription] to the primary care physician...and she says, 'I don't know what this is.' So then we'll send it to the neurologist and he goes, 'I don't know.' So then we have to find out who's the pulmonologist..."

### 3. Parents tracked their child's health information in their own comprehensive record as providers did not have this type of record

Some parents created notebooks organized with section dividers for various health information domains. Other parents created computer-based records by extracting important health information from copies of paper records obtained from their child's providers. Many parents created their own care plan summary in a single document containing a diagnosis and medication list, allergies, tracheotomy equipment settings and home nursing orders.

Parents and home care nurses used these summaries to facilitate communication with the children's providers and to order tracheotomy equipment and supplies. Parents reported that the creation and maintenance of their own comprehensive health record required considerable work, and felt that the effort required to assimilate, communicate, and share their child's health information with multiple providers was an unfair burden. They expressed a preference for providers taking responsibility for this task. Providers expressed mixed perceptions of the reliability of the information in a parent-created health record and their willingness to trust this information, particularly with regards to critical equipment information such as ventilator settings.

#### Parent

"We have our separate medical chart at home and a combination book that is circulated throughout that all the nurses read and that is sort of a daily update of what goes on with [our child]. It tracks when he goes into the doctor and what the doctor said. The home care nurses always have to have updated orders, signed change in orders from physicians, medical documentation and any kinds of medication change. So, that's always there at home for him. We have, for our own use, a daily record that narrates his day and any kind of changes made. So, it's both doctor documentation, his daily routine and what his day was like. It's all paper trail basically and of course anything that happens in the hospital or doctor's visits gets recorded and documented."

#### Tracheotomy Hospital Nurse

"Parents are absolutely fantastic with keeping [up with the] majority of [their children's medical information]. They have their own books. They handwrite everything in. They'll sit in the patient's room when the patient's in and they'll write down every single thing that happened. And the records they keep are phenomenal."

#### Parent

"Frankly it's beyond the doctor's door that's making my life hell. It's a lot of work to follow the paper trail; just a vigilant effort to push the process. The parents are probably the least able at some level because the work is necessary to keep our kids alive at home...It's grinding work. It doesn't take a lot of brain power, it just takes a lot of grueling vigilant effort and I get lost in it...There's so much [health information] that the parents get lost."

#### Respiratory Therapist

"I'm constantly looking at these kids to make sure that as inpatients, [we] set up the inpatient [ventilator] correctly because the parents quote you numbers, but the parents don't know, for example, for pressure support, if [they are providing] the dose or the number on their machine, which would be the highest number to pick. And that's a major [problem]. We hit that all the time."

### 4. Parents and providers expressed a need for an internet-accessible tracheotomy medical record that "floats" with the child through all medical encounters

Parents and providers did not want a record that resides within a single medical practice or hospital, but with the patient and family. Parents and providers described an ideal record as one that could be viewed, edited, shared and updated by all providers across multiple institutions. The ideal record would be internet-based and contain similar health information as in the parent or home nursing record.

Parents believed that this type of record could reduce the current burden of home record keeping for their children, especially with additional assistance. They felt that using the record could minimize the difficulties of retrieving, sharing and communicating their children's health information among the providers in various locations. Providers felt that using parents as the centralized "home" for the record could improve communication, care coordination and care plan development.

#### Parent of Child with Tracheotomy

"I've often thought that we have to get better about having some sort of a chart that goes with us... to have a better comprehensive record that goes from one place to another with him or if there is a way that hospitals could have [his] record flow from one hospital to another. [Hospital to] hospital communication is really difficult... It's sort of like you need some sort of record Blackberry."

#### Parent of a Child with Tracheotomy

"You need a secretary or personal assistant to help you keep [the record]."

#### Primary Care Physician

"The real answer is a patient-controlled, electronic medical record that lived on some sort of card that the patient carried with them or lived on some sort of government-run central database that all healthcare providers could access."

#### Otolaryngologist

"I think the ideal way of doing this is [to] somehow electronically transmit [clinical] notes to parents and they can download it onto a jump drive or something that they can bring with them to every visit; even if they're not capable of remembering all of the information, which [with] some of these kids you really can't. Everything is in an integrative [sic] electronic method that you can read it quickly through several different notes and review it. I don't think we're there yet and whoever solves that will probably end up solving a huge part of the healthcare problems in the country, not just for trach care."

### 5. Barriers to the development and use of a computerized record residing with patients and families for children with tracheotomy included record ownership, maintenance and control, information validity and "shadow file" attributes

Providers cited time-constraint as a barrier to enter health information into a child's personal health record, and were reluctant to enter information that other providers may change frequently, such as equipment settings. Parents and providers separately raised the issue that unless the record could become the ultimate "source of truth" for the children's health information, then its upkeep could be more of a burden than current methods, with little or no added clinical value.

#### General Pediatrician

"Who has got time to write [tracheotomy information] into an internet record? What I really ought to do is I ought to invite the parents or somebody who deals with the kid to write that stuff and I'll put it in the chart."

#### General Pediatrician

"The problem I have found is with ventilator settings. You don't want to lock them down in a record and then have [another provider] change the settings, causing your record to have incorrect information."

#### Nurse Practitioner

"The big problem is the credibility of this type of [the health information in the record]. It's like the kid that comes from outside to your emergency department and has them send a lab. You're like, 'I don't believe these labs. I'm repeating them myself.' You know, it's like you're going to look at [the record] and say, 'how do I know that it's actually accurate?' It's not all my information. I can't swear that it's all accurate and true."

#### General Pediatrician

"Am I going to prune [the otolaryngology care plan]? What am I going to do [when] the parents tell me the kid is currently on [medication A], but the otolaryngologist wrote that should they really be on [medication B]?"

#### General Pediatrician

"[This] struck me as being a shadow chart. And shadow charts I've been taught in all my risk management things... [that] they're a risk because a lawyer can find discrepancies between a shadow chart and a real chart and they can hang you with them. And so I was always taught that you don't keep shadow charts and this seemed like a shadow chart to me."

#### General Pediatrician

"If you spend all this time and money and programming to do this thing and at the end of the day, if you saw what you thought was the source of truth, but you didn't completely trust it, and you're still calling the pulmonologist, then you're right where you are now... It becomes yet another source of information that you technically become responsible for - like looking at and seeing... it's just more work than helpful."

## Discussion

This study reveals disquieting descriptions of health information management and perceptions of its impact on the quality of care for children with tracheotomy who participated in this study. The findings suggest a health system ecology among study participants characterized by poorly defined provider roles and responsibilities, inadequate communication, delays and omissions in care information. Parents reported creating a home record that they shared with multiple providers to track their children's care. Parents considered this an unfair burden better suited to providers. Providers benefited from using the parent records, but questioned their accuracy regarding critical tracheotomy care plan information such as ventilator settings. Parents and providers endorsed potential improvement in this environment such as a comprehensive internet-based health record that could be shared among parents and providers, and accessed at multiple clinical sites.

The development of an internet-based comprehensive health record for children with tracheotomy that includes all health information within one central location is unlikely in the near future without rapid integration and inter-operability improvements among hospitals, community and health records kept by parents. However, this study outlines a series of small scale strategies and testable hypotheses that will inform the development and evaluation of both short and longer-term solutions to improve health information management and quality of care across healthcare sectors for these children. These strategies and hypotheses are discussed below.

One hypothesis that could be generated from this study is that establishing tracheotomy care delivery roles and responsibilities among providers would improve care team operations and information exchange for patients in our study. Clearly-defined roles and responsibilities are described as a consistent attribute of an effective care team and they have been associated with improved patient outcomes [26-30]. They can help overcome uncertainty regarding division of clinical responsibilities (e.g., a home nurse who is uncertain which provider is responsible for overseeing home care orders) and information management (e.g., an otolaryngologist who is uncertain which provider is responsible for maintaining current tracheotomy equipment information) described by participants in this study [31,32].

As improved roles and responsibilities among a child's tracheotomy care team are evaluated, it will be important to consider strategies that will assist and support parents in our study who were the primary health information managers for their children. Parents of children with other complex medical conditions report this phenomenon, and hospital and community-based programs for CSHCN encourage families to keep their own records [33,34]. Some parents suggested that they would benefit from a person who helps maintain their home-based records. There may be personnel options for record management assistance beyond physicians who are subject to time-constraints. Studies carried out in adults report patient favorability for health coaches (e.g., case managers or care coordinators) that help facilitate health information transfer and maintenance during hospitalization and after hospital discharge [35].

Web-conversion of parents' paper and word processed tracheotomy home records is another strategy that could reduce parents' health information management burden. There are several software applications including GoogleDoc and Dropbox, which support web-based document viewing, editing and sharing among multiple users [36,37]. These applications could allow parents to enter and update their child's health information into a web-based document from any computer with internet access, and share it electronically with their child's health care providers. With parental permission, multiple providers from various settings can view and update the document. Further evaluation is required to test the hypothesis that web-based conversion decreases the effort and time required to maintain and share a tracheotomy home record.

Web-based personally-controlled health records (PCHRs) are an additional strategy that may enhance the functionality of home records. PCHRs allow patients and caregivers to enter disease-specific information directly into the record and they are capable of receiving health record information directly from existing electronic health record sources [38]. PCHRs enable individuals to aggregate, securely store and access electronic health information from multiple sites, and to share that information with care providers [39,40]. PCHR prototypes are currently under testing for use in children with chronic conditions [38]. PCHR for children with tracheotomy could be a critical step in bridging the inter-operability of parent-kept records and existing EHRs across multiple healthcare sectors.

Perceived barriers of a web-based tracheotomy health record in our study were information trust and duplication, and further investigation is required to test strategies that could overcome these barriers. For instance, providers may be more likely to trust tracheotomy health information that has been authenticated, such as displayed home nursing orders with an electronic signature from the provider in charge. Existing hospital and ambulatory EHRs offer this capability with electronic prescription writing and order entry [41]. It may be possible to adapt this capability into developing web-based records. Information duplication may be minimized by presenting data within a web-based record that is perceived as frequently lost or mismanaged among tracheotomy providers' existing records, such as tracheotomy equipment settings. A web-based record may be suited to primarily host and display this information if it can be uniformly formatted, summarized, verified and updated easily.

There are several limitations to the present study. The sample size is small, with a limited number of participants from each provider type and this precluded ascertaining the strength or prevalence of the observed perceptions and experiences. However, the themes evoked by the qualitative findings could inform future investigations concerning health information management with a quantitative survey given to a larger sample of children with tracheotomy that focuses on roles and responsibilities among tracheotomy providers and various strategies to help parents maintain home-based health records.

The perceptions and experiences in this study were derived from information-rich cases and are not intended to be representative of all parents and providers of children with tracheotomy. There are likely to be local and regional healthcare system attributes specific to the study setting which may influence the findings. There may be additional important experiences and perceptions not captured by the study. For instance, the perceptions of health care reform initiatives that reward tracheotomy providers for care coordination activities that facilitate improved health information exchange among them and their patients (e.g., helping families update and maintain home-based records) were not explored. Further investigation is required to determine if tracheotomy outpatient providers would be more willing to participate in these activities if they are held financially accountable for them or if payment incentives, such as improved fee-for-care coordination services, are present [42].

## Conclusions

Participants in the present study described important situations relating to disorganized tracheotomy care and mismanagement of health information that may help guide future investigations into the impact of improved health information systems for children with tracheotomy. Strategies with the potential to improve tracheotomy care delivery include improved tracheotomy providers' roles and responsibilities and better organization and improved parent support to maintain home-based tracheotomy records with web-based software applications, personal health record platforms and health record data authentication techniques. There is a critical need to test whether these strategies improve tracheotomy health information management and exchange, and if they contribute to improved quality of care and health outcomes in children with tracheotomy.

## Competing interests

The authors declare that they have no competing interests.

## Authors' contributions

JGB and HP participated in the design of the study, recruited, enrolled and interviewed participants, coded their data, and drafted the manuscript. RW participated in the design of the study, helped code data and drafted the manuscript. DG, KM, DH and JO participated in the design of the study and helped draft the manuscript. All authors have read and approved the final manuscript.

## Pre-publication history

The pre-publication history for this paper can be accessed here:

http://www.biomedcentral.com/1472-6963/11/117/prepub

## Supplementary Material

Additional file 1 **Table S1. Individual and Group Interview Questions**. The list of questions asked in the individual and group interviews. Click here for file
