# Supplementary material for: Health information management and perceptions of the quality of care for children with tracheotomy: A qualitative study
Source: BMC Health Serv Res. 2011 May 23;11:117. doi: 10.1186/1472-6963-11-117 (PMC3127978; doi:10.1186/1472-6963-11-117)
Supplement: Additional file 1 — Table S1. Individual and Group Interview Questions. The list of questions asked in the individual and group interviews. [file 1472-6963-11-117-S1.DOC]

**Table S1. Individual and Group Interview Questions**

| **Parent Schedule** |
| --- |
| Please tell me what each of (child’s name)’s providers are responsible for in regard to his care. |
| Please tell me about a time when (your child’s) healthcare providers worked well together. |
| Why did they work well together? |
| What about a time when they did not work well together? |
| Why did they not work well together? |
| If a provider makes a change in (child’s name)’s care plan then how do you find out that change has occurred? |
| How is that change communicated to his/her other healthcare providers? |
| How does this influence the care your child receives? |
| Please tell me about (child’s name)’s health records and his tracheotomy care plans. |
| Who is in charge of the information? |
| Who updates the information? |
| Where is the information stored? |
| Is it readily-available to you and (child’s name)? |
| How is (child’s name)’s health influenced by having (or not having) a plan in place? |
| How do you keep up with the (child’s name)’s tracheotomy-related health information? |
| How could we improve the care that (child’s name)’s receives? |
| How could we make (child’s name)’s providers work better together for (child’s name)’s health? |
| **Provider Schedule** |
| Please tell me what each provider is responsible for in regard to care for a child with tracheotomy. |
| Please tell me about a time when you collaborated or worked closely with other healthcare providers to care for a child with a tracheotomy. |
| Why did you work well together? |
| What about a time when you did not work well together with other providers? |
| Why did you not work well together? |
| If a provider makes a change to a child’s care plan then how do you find out that change has occurred? |
| How is that change communicated among the healthcare providers? |
| How does this influence the care the children receive? |
| Please tell me about the health records and care plans for children with tracheotomy in your practice. |
| How do you keep up with the tracheotomy-related health information? |
| Who is in charge of the information? |
| Who updates the information? |
| Where is the information stored? |
| Is it readily-available to you? |
| How is the children’s health influenced by having (or not having) a plan in place? |
| How could we improve the care that children with tracheotomy receive? |
| How could we make their providers work better together? |
